# Bloodstream Infection among Adults in Phnom Penh, Cambodia: Key Pathogens and Resistance Patterns

**DOI:** 10.1371/journal.pone.0059775

**Published:** 2013-03-29

**Authors:** Erika R. Vlieghe, Thong Phe, Birgit De Smet, Heng Chhun Veng, Chun Kham, Kruy Lim, Olivier Koole, Lut Lynen, Willy E. Peetermans, Jan A. Jacobs

**Affiliations:** 1 Department of Clinical Sciences, Institute of Tropical Medicine, Antwerp, Belgium; 2 Sihanouk Hospital Centre of HOPE, Phnom Penh, Cambodia; 3 Department of Internal Medicine, University Hospital, Leuven, Belgium; Oxford University, Viet Nam

## Abstract

**Background:**

Bloodstream infections (BSI) cause important morbidity and mortality worldwide. In Cambodia, no surveillance data on BSI are available so far.

**Methods:**

From all adults presenting with SIRS at Sihanouk Hospital Centre of HOPE (July 2007–December 2010), 20 ml blood was cultured. Isolates were identified using standard microbiological techniques; antibiotic susceptibilities were assessed using disk diffusion and MicroScan®, with additional E-test, D-test and double disk test where applicable, according to CLSI guidelines.

**Results:**

A total of 5714 samples from 4833 adult patients yielded 501 clinically significant organisms (8.8%) of which 445 available for further analysis. The patients’ median age was 45 years (range 15–99 y), 52.7% were women. HIV-infection and diabetes were present in 15.6% and 8.8% of patients respectively. The overall mortality was 22.5%. Key pathogens included *Escherichia coli* (n = 132; 29.7%), *Salmonella* spp. (n = 64; 14.4%), *Burkholderia pseudomallei* (n = 56; 12.6%) and *Staphylococcus aureus* (n = 53; 11.9%). Methicillin resistance was seen in 10/46 (21.7%) *S. aureus*; 4 of them were co-resistant to erythromycin, clindamycin, moxifloxacin and sulphamethoxazole-trimethoprim (SMX-TMP). We noted combined resistance to amoxicillin, SMX-TMP and ciprofloxacin in 81 *E. coli* isolates (62.3%); 62 isolates (47.7%) were confirmed as producers of extended spectrum beta-lactamase. *Salmonella* isolates displayed high rates of multidrug resistance (71.2%) with high rates of decreased ciprofloxacin susceptibility (90.0%) in *Salmonella* Typhi while carbapenem resistance was observed in 5.0% of 20 *Acinetobacter* sp. isolates.

**Conclusions:**

BSI in Cambodian adults is mainly caused by difficult-to-treat pathogens. These data urge for microbiological capacity building, nationwide surveillance and solid interventions to contain antibiotic resistance.

## Introduction

Sepsis is worldwide associated with important morbidity and mortality [1] with bloodstream infection (BSI) as one of its main causes. Early administration of adequate antibiotic therapy is essential to improve patient outcomes [2] and should be based on accurate knowledge of local bacterial pathogens and their resistance patterns. However, the worldwide emergence of antibiotic resistance has led to drastic changes in the choice of antibiotics for empiric or directed treatment in national or international treatment guidelines, with an evolution towards increased use of broad spectrum antibiotics.

Asia, and in particular its Southeast and East Asia regions are struck profoundly by the problem of antimicrobial resistance [3]. Its extent has been described well in the regions’ high and middle income countries, whereas accurate data and in depth research in low resources settings such as Cambodia are scarce.

Sihanouk Hospital Centre of HOPE (SHCH) is a Non-Governmental Organization (NGO) hospital for adults in Phnom Penh, Cambodia. It provides free care for the poor with specific focus on patients with the human immunodeficiency virus (HIV) and the chronically ill. Yearly, care is given to an average of 6000 ambulatory and 1200 hospitalized patients. Microbiological facilities were installed in 2005, along with a local capacity building program focusing on diagnosis and management of antibiotic resistance at hospital level. In July 2007, a prospective blood culture study was initiated in patients presenting with presumed BSI. The primary objective of this study was to determine the key bacterial pathogens and their resistance patterns causing invasive infections in Cambodian adults. As a secondary objective the information retrieved would be used for the redaction of locally adapted standard treatment guidelines. This would also include an exploration of possible risk factors for BSI and particular key pathogens. In this article we present the findings of this study.

## Materials and Methods

### Patient Selection and Microbiological Methods

From all adult patients presenting at SHCH with signs of the Systemic Inflammatory Response Syndrome (SIRS) [4], venous blood (2×10 ml) was drawn for culture along with registration of basic demographic and clinical data (including sex, age, duration of hospitalization, recent use of antibiotics, co-morbidity, presenting signs and symptoms and presumed focus of infection.).

Blood was cultured in home-made Brain Heart Infusion broth bottles (BIO-RAD, Hercules, US; 50 ml per bottle, July 2007-March 2009), and from April 2009 onward in BacTalert culture bottles (bioMérieux, Marcy l’Etoile, France). Blood cultures were incubated for 7 days at 35°C and daily monitored for growth by visual inspection of the broth or the chromogenic growth indicator where applicable. Isolates were stored at −70°C on porous beads in cryopreservative (Microbank, Pro-Lab Diagnostics, Richmond Hill, Canada). As part of the study protocol, isolates were retrieved and identification tests and antimicrobial susceptibility testing by disk diffusion were repeated at the Institute of Tropical Medicine, Antwerp (ITM, Belgium).

Isolates were identified using standard microbiological techniques and Micro**S**can®(Combo 42, Siemens Healthcare Diagnostics, Deerfield, USA). *Salmonella* spp. were serotyped according to the Kauffman-White schedule [5]. Antibiotic susceptibilities were assessed by disk diffusion (Neo-Sensitabs™, Rosco Diagnostica, Taastrup, Denmark) and Micro**S**can®(microdilution method). Additional testing, where applicable, included E-test (bioMérieux, for determination of penicillin minimal inhibitory concentrations (MIC) in *Streptococcus pneumoniae)*, D-test [6] for the detection of inducible clindamycin resistance in *Staphylococcus aureus* and double disk test with ceftazidime, cefepime, ceftazidime-clavulanic acid and cefepime-clavulanic acid as described elsewhere [7] (for extended spectrum β-lactamase-screening in *Enterobacteriaceae*). Interpretive criteria were those defined by the Central Laboratory Standards Institute [8] except for fosfomycin and colistin testing in *Enterobacteriaceae* and *Pseudomonas aeruginosa* where MicroScan® refers to EUCAST breakpoints. For azithromycin and *Enterobacteriaceae*, no breakpoints have been published. EUCAST mentions treatment of *Salmonella* Typhi infections with a MIC≤16 µg/mL and a recent publication proposed 16 µg/ml as ‘epidemiological cut-off’ value for wild type *Salmonella* spp [9]. The following isolates were considered as contaminants: coagulase-negative *Staphylococci*, *Bacillus* spp., *Corynebacterium* spp. Non-eubacterial pathogens (e.g. mycobacteria and fungi) were left out of the analysis.

The minimum number of isolates per species for separate antibiotic resistance reporting was set at 10, in line with the recommendations of the ESCMID Study Group for Antimicrobial Resistance Surveillance (ESGARS) [10] and of the U.S. Clinical and Laboratory Standards Institute (CLSI) at the time of the initiation of the study (i.e. CLSI M2-18).

For the assessment of antimicrobial susceptibility rates, only the first isolate per patient was considered. Growth from a repeat blood culture sampled more than 14 days after the previous one in a patient on adequate antibiotic treatment was considered as a new BSI episode. Recurrent isolates (i.e. same species isolates from different BSI episodes) and consecutive isolates (i.e. different species’ isolates from different BSI episodes) were not compiled in the resistance overview.

Isolates from patients who were not hospitalized or admitted no more than two days on the moment of blood culture sampling were considered community-acquired whereas isolates from patients hospitalized longer than two days were considered hospital-acquired. ‘Shock’ was defined as presenting with a mean arterial pressure (MAP) of <60 mmHg despite adequate intravenous fluid resuscitation [4]. Fever and cough were considered ‘acute’ if less than three weeks of duration and ‘chronic’ if persisting for ≥3 weeks.

The period between November 16^th^ and May 15^th^ was considered the dry season, and May 16^th^-November 15^th^ the wet season.

### Data Registration and Statistical Analysis

Data were entered in Access and Excel databases (Microsoft Corporation, Redmond, Washington, USA). Stata software, version 11.2. (Stata Corporation, College Station, TX, USA) was used for statistical analysis. Risk factors for BSI and particular key pathogens were explored using univariate analysis (chi-square tests). Differences were considered statistically significant at p-values <0.05.

### Ethical Approval

Ethical approval was granted from the review boards at the Institute of Tropical Medicine (ITM), Antwerp, the University Hospital Antwerp and the National Ethical Committee, Phnom Penh, Cambodia respectively. A waiver for prior informed consent was obtained since samples were taken as part of routine clinical care. Patients were identified with a unique hospital number. For the clinical and epidemiological data, no other data besides those noted in the routine medical files were used.

## Results

### Demographical and Epidemiological Data

**Figure 1:**
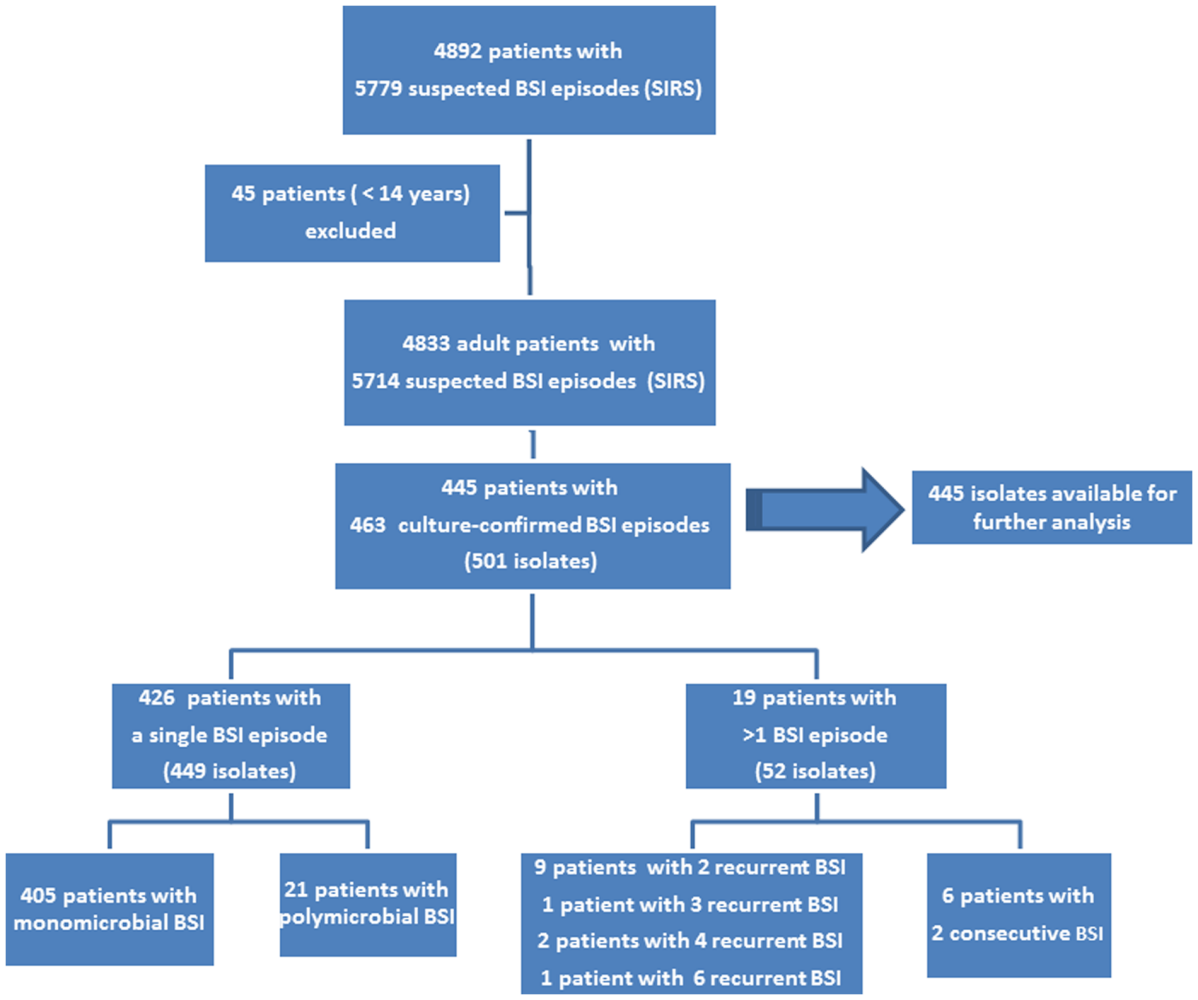
Flow chart of patients with SIRS episodes and corresponding episodes of bloodstream infection. SIRS was defined as the presence of more than one of the following clinical findings: body temperature of >38°C or <36°Celsius, heart rate >90 beats per minute, respiratory rate >20 per minute, PaCO_2_<32 mmHg, whit e blood cell count >12000 cells/µL or <4000 cells/µL [4].

Between July 1^st^ 2007 and December 2^nd^ 2010, a total of 4833 adult patients with SIRS attended the hospital and had blood cultures drawn during 5714 SIRS episodes (Figure 1). Fifty-three per cent of the patients were women, with a median age of 45 years (15–99 y)(Table 1). Patients came from at least 14 different provinces in Cambodia, predominantly from the Central and South of the country. Co-morbidity was noted in nearly one third (1579/4833; 32.7%) of the patients, mainly HIV-infection (15.6%) or diabetes mellitus (8.8%). Eighty-six per cent (4912/5714) of the SIRS episodes were community-acquired, while 370 of them (6.5%) were considered hospital-acquired. Recent use of antibiotics prior to blood culture sampling was noted in 1270 episodes (22.2%).

**Table 1:** Demographic and clinical characteristics of patients with SIRS and BSI (SHCH, 2007–2010).

		all SIRS-episodes		culture confirmed BSI-episodes	
		n	%	n	%
Total number of patients		4833		445	
Female		2547	52.7	226	50.8
Median age (range)		45 (15–99)		46 (15–96)	
Co-morbidity	HIV-positive	755	15.6	82	18.4
	diabetes	425	8.8	51	11.5
	liver cirrhosis	196	4.1	25	5.6
	other*	286	5.9	23	5.2
Total number of episodes		5714		463	
Hospitalisation**	Not or ≤2 days	4912	86.0	404	87.3
	>2 days	370	6.5	48	10.4
	No data	423	7.4	46	9.9
Recent antibiotic treatment		1270	22.2	112	24.2
Presumed focus***	abdominal	2049	35.9	207	44.7
	respiratory	1090	19.1	152	32.8
	urogenital	576	10.1	64	13.8
	SSTI	480	8.4	47	10.2
	meningeal	348	6.1	34	7.3

SIRS: systemic inflammatory response syndrome; BSI: blood stream infection; HIV: human immune deficiency virus; SSTI: skin and soft tissue infections.

* includes chronic lung or renal disease, chronic use of steroids.

** refers to duration of hospitalisation at moment of blood culture sampling.

*** in some patients more than one presumed focus was noted.

Acute fever (n = 4117; 72.1%) was the most common presenting symptom followed by acute cough (n = 445; 7.8%), chronic fever (n = 211; 3.7%), diarrhoea (n = 196; 3.4%), chronic cough (n = 176; 3.1%), dysuria (n = 176; 3.1%), meningism (n = 140; 2.5%) and shock (n = 100; 1.8%). Abdominal, respiratory and urogenital infections were the most common presumed foci of BSI (Table 1).

### Microbiological Data

In four hundred and sixty-three SIRS episodes, 501 clinically significant organisms (CSO) were cultured (yield 8.8%). In addition, a total of 496 contaminants (contamination rate 8.7%) were isolated. The CSO yield in samples from patients with prior antibiotic use (112/1270; 8.8%) was not different from patients without known antibiotic use (389/4440, 8.8%).

A total of 445 CSO were available for further analysis. Of those, 404 (90.8%) were isolates from community-acquired infections and 41 (9.2%) were hospital-acquired.

As shown in Table 2, Gram negative pathogens were predominant (351/445; 78.9%). The most frequent pathogens overall included *Escherichia coli* (n = 132), *Salmonella* spp. (n = 64), *Burkholderia pseudomallei* (n = 56) and *Staphylococcus aureus* (n = 53). Hospital-acquired BSI were mainly caused by *S. aureus* (5/41; 12.2%), *E. coli* and other *Enterobacteriaceae* (21/41; 51.2%) and *Acinetobacter* spp. (7/41; 17.1%). Upon retesting at ITM, we observed genus identification agreement in 98.4% of isolates; a particular species identification disagreement was noted for the first isolates of *Streptococcus suis* (n = 3) and *Salmonella Choleraesuis* (n = 17) which were erroneously identified as *Streptococcus pneumoniae* and *Salmonella* Paratyphi A/Salmonella species respectively.

**Table 2:** Pathogen distribution in 445 blood culture isolates, SHCH 2007–2010.

Species	total n (%)	n HA isolates^1^	n first BSI's isolates^2^
**Gram positive cocci**	94		
*Staphylococcus aureus*	53 (11.9)	5	46
β-hemolytic streptococci group A	2 (0.4)	–	–
group B	2 (0.4)	–	–
group C	4 (0.9)	–	–
group G	1 (0.2)	–	–
*Streptococcus pneumoniae*	11 (2.5)	2	11
*Streptococcus suis*	9 (2.0)		–
*Streptococcus anginosus* group	2 (0.4)	1	–
*Streptococus bovis*	2 (0.4)	–	–
*Streptococcus salivarius*	2 (0.4)	–	–
*Enterococci* spp^3^	6 (1.3)	–	–
**Gram negative bacilli**			–
**Enterobacteriaceae**	351		–
*Escherichia coli*	132 (29.7)	13	130
*Klebsiella pneumoniae*	34 (7.6)	5	32
*Enterobacter* spp.	14 (3.1)	3	13
*Proteus* spp.	5 (1.1)	–	–
*Aeromonas* spp^4^	11 (2.5)	–	11
*Shigella* spp.	2 (0.4)	–	–
*Vibrio* cholerae	1 (0.2)	–	–
*Citrobacter* sp.	1 (0.2)	–	–
*Salmonella* Typhi	15 (3.4)	1	15
*Salmonella* paratyphi A	2 (0.4)	–	–
non -typhoid *Salmonella* spp. (NTS)		–	–
serovar Choleraesuis	35 (7.9)	1	23
serovar Enteritidis	6 (1.3)	–	–
serovar Typhimurium	4 (0.9)	–	–
other NTS	2 (0.4)	1	–
**non-fermentative Gram negative rods**	87	–	–
*Burkholderia pseudomallei*	56 (12.6)	1	56
*Acinetobacter* spp^5^	20 (4.5)	7	20
*Pseudomonas* spp^6^	8 (1.8)	–	8
other	3 (0.7)	1	–
Total	445	41	–

1 HA: hospital acquired isolates;

2 isolates used for calculation of resistance rate;

3 includes E. faecium (n = 3), E. fecalis (n = 2), E. raffinossus (n = 1);

4 includes A. hydrophilia group (n = 8), A. species (n = 1), A.sobria (n = 1);

5 includes A. baumanii/haemolyticus (n = 17), A. lwoffi (n = 3);

6 includes P. aeruginosa (n = 2), P. stutzeri (n = 5), P. fluorescens/putida (n = 1).

In 426 patients a single BSI episode was noted whereas nineteen patients experienced multiple episodes of BSI (Figure 1). Of those patients, thirteen had genuine recurrent BSI (i.e. with the same species), most commonly caused by *Salmonella* Choleraesuis (a total of 17 episodes in seven patients), *S. aureus* (seven episodes in three patients) and *E. coli* (three patients with two episodes each). Six other patients had consecutive episodes of BSI (i.e. with different species). Only the isolates of the first BSI episodes were taken into account for the calculation of the key pathogens’ resistance rates (Table 2).

BSIs were generally more frequent in patients with diabetes (59/494 (11.9%) versus 442/5220 (8.5%) patients without diabetes, p = 0.009) and during the wet season (299/3132 (9.5%) versus 202/2582 (7.8%) episodes during the dry season, p = 0.020). *E coli* BSI occurred more frequently in patients aged ≥60 years (37/1066 (3.5%) versus 90/4567 (2.0%) patients <60 years old, p = 0.003) and in those with diabetes (20/494 (4.0%) versus 112/5220 (2.1%) patients without diabetes, p = 0.007), whereas *B. pseudomallei* BSI was seen more often in men (40/2685 (1.5%) versus women 16/3006 (0.5%), p<0.005) and diabetes patients (14/494 (2.8%) versus 42/5220 (0.8%) in those without diabetes, p<0.005). In contrast, *Salmonella* BSI occurred more often in HIV-positive patients (37/1186 (3.1%) versus 30/4528 (0.7%) HIV-negative patients, p<0.005) as was also the case for *S. aureus* BSI (17/1186 (1.4%) HIV-positive patients versus 36/4528 (0.8%) HIV-negative patients, p = 0.041).

Out of 501 BSI patients, 330 (65.9%) recovered, 113 died (22.5%), 25 (4.9%) were referred to another hospital, 33 (6.6%) had an unknown outcome. The median outcome follow-up time was 138 days (range 0–1982 d); death occurred after a median duration of 2 days of BSI diagnosis (range 0–62 d). Mortality was higher than average in patients with BSI due to *B. pseudomallei* (29/55; 52.7%) and *E. coli* (35/131; 26.7%), and lower in those with BSI due to *S. aureus* (9/53; 17%) and *Salmonella* spp. (17/66; 10.6%). In addition, we found higher mortality rates in patients with BSI due to third generation-cephalosporin resistant *E. coli* (20/50 (40.0%) versus 15/65 (23.1%) in those without this resistance pattern) and for patients with methicillin susceptible *S. aureus* (MSSA, 8/38 (21.1%) versus those with methicillin resistant *S. aureus* (MRSA) BSI (1/8; 12.5%), but both findings were not statistically significant. Prior use of antibiotics influenced significantly the presence of third generation-cephalosporin resistance in *E. coli* BSI (15/19 (79.0%) versus 46 of 112 (41.9%) patients without prior antibiotic treatment; p = 0.002), but it did not significantly affect the overall patients’ mortality (21/103 (20.4%) versus 92/340 (27.1%) in those without prior antibiotic use; p = 0.20).

### Gram Positive Cocci

Table 3 displays the antibiotic resistance of methicillin resistant *S. aureus* (MRSA) and methicillin susceptible *S. aureus* (MSSA). MRSA was present in 10 of 46 *S. aureus* (21.7%) from first BSI episodes and was associated with high levels of resistance to clindamycin, moxifloxacin, erythromycin and tetracycline. Four MRSA isolates (25.0%) displayed combined resistance to erythromycin, clindamycin, SMX-TMP, moxifloxacin and tetracycline. This co-resistance was also noted to a lesser extent in MSSA (2/36 isolates (5.6%)). Glycopeptide susceptibility was preserved. The presence of Methicillin resistance in *S. aureus* BSI was not significantly more prevalent in patients with prior antibiotic use (3 of 12 (25.0%) than among those without antibiotic pre-use (7/41 (17.0%), p = 0.70).

**Table 3:** Antibiotic resistance patterns of 46 *S. aureus* from blood, SHCH 2007–2010.

	MRSA	MSSA
Antibiotic	(n = 10)	(n = 36)
Penicillin	100	97.2
Erythromycin	90.0	41.7
Clindamycin*	100	38.9
Tetracycline	90.0	41.7
SMX-TMP	60.0	13.9
Moxifloxacin	90.0	30.6
Vancomycin	0.0	0.0
Fusidic acid	10.0	5.6

* including inducible clindamycin resistance.

SMX-TMP: sulphamethoxazole-trimethoprim; MRSA: methicillin resistant Staphylococcus aureus; MSSA: methicillin susceptible Staphylococcus aureus.

In the 11 *S. pneumoniae* isolates, a median MIC penicillin of 0.12 µg/ml (range 0.012–4 µg/ml) was noted, which translates according to the 2012 CLSI guidelines [8] as intermediate and high level resistance to oral penicillins in 5 and 2 isolates respectively, and as intermediate resistance to parenteral penicillins in one isolate for non-meningitis patient. Resistance for SMX-TMP, ceftriaxone, and erythromycin was noted in 8/11(72.7%), 0/11 (0%) and 2/11(18.2%) of the isolates.

### Enterobacteriaceae

As shown in Table 4, *E. coli* and other *Enterobacteriaceae* were extensively resistant to all commonly available oral first line antibiotics (i.e. ampicillin, SMX-TMP and ciprofloxacin) and gentamicin. Nearly half of all *E. coli* (47.7%) were confirmed extended spectrum β-lactamase (ESBL) producers. *Enterobacter* spp. displayed also considerable resistance rates for reserve antibiotics (e.g. piperacillin-tazobactam, tigecyclin, colistin, amikacin).

**Table 4:** Antibiotic resistance patterns of *Enterobacteriaceae* from blood, SHCH 2007–2010.

	*E. coli*	*Klebsiella* spp.		*Enterobacter* spp.
	(n = 130)	(n = 32)		(n = 13)
Antibiotic	% resistant isolates			
Ampicillin	93.8	100,0	100,0	
Amoxicillin/clavulanic acid	49.2	28.1	84.6	
SMX-TMP	95.4	75.0	76.9	
Ciprofloxacin	65.4	21.9	46.2	
Cefotaxime	51.5	46.9	61.5	
Ceftazidime	36.2	25.0	46.2	
Cefepime	46.2	28.1	46.2	
ESBL confirmed*	47.7	43.8	46.2	
Gentamicin	56.2	28.1	38.5	
Amikacin	3.8	0.0	7.7	
			23.1	
Piperacillin/tazobactam	9.2	15.6	23.1	
Meropenem	0.0	0.0	0.0	
Colistin	0.8	3.1	53.8	
Tigecycline	0.0	6.3	15.4	
Fosfomycin	0.0	15.6	38.5	

* by double disk testing.

SMX-TMP: sulphamethoxazole-trimethoprim; ESBL: extended spectrum β-lactamase.

In addition, one third of all *Enterobacteriaceae* (58/175) were co-resistant for the most commonly available oral or parenteral antibiotics in Cambodia (i.e. ampicillin, SMX-TMP, ciprofloxacin, gentamicin and third generation cephalosporins). Co-resistance to amikacin was noted in 3 of 175 (1.7%) isolates (Table 5).

**Table 5:** Combined resistance in *Enterobacteriaceae* from blood, SHCH 2007–2010.

	*E. coli*	*Klebsiella* spp.	*Enterobacter* spp.
	(n = 130)	(n = 32)	(n = 13)
Antibiotic	% resistant isolates		
Ampicillin+SMX-TMP+ciprofloxacin (AmSxCip)	62.3	18.8	46.2
AmSxCip+gentamicin	47.7	12.5	30.8
AmSxCip+gentamicin+cefotaxime	38.5	12.5	30.8
AmSxCip+gentamicin+cefotaxime+amikacin	2.3	0.0	0.0

SMX-TMP: sulphamethoxazole-trimethoprim.

The resistance patterns of *Salmonella* spp. from this BSI study were described separately [11]. We observed high rates of multidrug resistance (*i.e.* co-resistance to ampicillin, SMX-TMP and chloramphenicol) in 75.0% of *Salmonella* Typhi, 91.7% of *Salmonella* Choleraesuis and in 38.5% of other non-typhoid *Salmonella* spp. (NTS). Decreased ciprofloxacin susceptibility was very common in *Salmonella* Typhi (14/15 isolates, 90.0%) and somewhat less frequent in *Salmonella* Choleraesuis (20.8%) and other NTS (53.8%) while azithromycin resistance was very common in S*almonella* Choleraesuis (17/24 isolates, 70.8%) and emerging in other NTS (15.4%) and *Salmonella* Typhi (5.0%). Two *Salmonella* Choleraesuis isolates were extended spectrum beta-lactamase producers. One NTS isolates displayed high level ciprofloxacin resistance (MIC 6 µg/ml).

In addition to their natural resistance for ampicillin, most *Aeromonas* isolates were largely resistant for amoxicillin-clavulanic acid (10/11; 90.9%) and to a lesser extent for cefotaxime (1/11; 9.0%), SMX-TMP and meropenem (2/11, 18.2% each) but all remained fully susceptible for ciprofloxacin.

### Non-fermentative Gram Negative Rods

As we described elsewhere [12], we did not observe *Burkholderia pseudomallei* isolates resistant for ceftazidime, meropenem, amoxicillin/clavulanic acid or doxycycline, but 12 (22.2%) isolates had MIC’s equal to the susceptibility breakpoint for chloramphenicol. *B. pseudomallei* has intrinsic resistance for amoxicillin, aminoglycosides and polymyxins while fluoroquinolones have only weak clinical efficacy [13].

High resistance levels for most available effective drugs and emerging carbapenem resistance were observed in other non-fermentative Gram negative rods (i.e. particularly *Acinetobacter* and *Pseudomonas* spp. (Table 6). Of note, *Acinetobacter* sp. was found to be of hospital-acquired origin in at least 7 of 20 (35%) isolates.

**Table 6:** Antibiotic resistance patterns of non-fermentative Gram-negative rods from blood, SHCH 2007–2010.

	*Acinetobacter* spp.			*Pseudomonas* spp.		
Antibiotic	A. baumanii (n = 17)	A. lwoffi (n = 3)		P. aeruginosa (n = 2)	P. non-aeruginosa (n = 6)	
	n resistant isolates (%)			n resistant isolates		
SMX-TMP	13 (76.5)	1	2		4	
Ciprofloxacin	7 (41.2)	2	0		1	
Gentamicin	10 (58.8)	0	0		0	
Amikacin	7 (41.2)	0	0		0	
Ceftazidime	9 (52.9)	0	0		0	
Cefepime	9 (52.9)	0	0		1	
Piperacillin/tazobactam	0 (0.0)	0	0		0	
Meropenem	1 (5.9)	0	0		1	
Colistin	3 (17.6)	0	0		1	

SMX-TMP: sulphamethoxazole-trimethoprim;

## Discussion

In our study we observed a predominance of Gram negative pathogens causing BSI in Cambodian adults; the three most frequent pathogens were *E. coli*, *Salmonella* spp., *B. pseudomallei*, followed by *S. aureus*. Many of these pathogens showed resistance to the antibiotics which are commonly available in Cambodia either by natural or by acquired resistance (e.g. *B. pseudomallei* and ESBL-producing bacteria respectively). In particular, resistance rates are alarming in *Enterobacteriaceae* displaying third generation cephalosporin resistance in up to 50% of isolates and nearly 1 in 3 isolates displaying complex combined resistance leaving very few treatment options.

One of the primary strengths of our findings is the fact that they were derived from systematically collected bloodstream isolates and obtained through a ‘real live’ capacity building program, where we observed excellent identification correlations between the laboratories of SHCH and ITM.

For the local health care workers, these data functioned as an ‘eye opener’ regarding the incidence in Cambodia of certain pathogens and specific resistance patterns (e.g. *B. pseudomallei*, ESBL-positive *Enterobacteriaceae*, *Streptococcus suis*, *Salmonella* Choleraesuis). They were also the basis of a better communication between the laboratory and the clinicians at SHCH and initiated a set of antibiotic stewardship activities. These included educational sessions, the development and implementation of standard treatment guidelines, the creation of a hospital essential drug list, enhanced communication with antibiotic donation agencies and finally collaboration with initiatives at national level.

Our study had also several limitations. First, the high rates of co-morbidity in our study population may have caused over-representation of specific pathogens (e.g. *B. pseudomallei* in diabetic patients, *Salmonella* Choleraesuis in patients with HIV). Therefore these findings should be interpreted in the context of these specific patient groups. However, given the fast evolution of the Asian diabetes epidemic and the on-going HIV-epidemic, rising numbers of these particular invasive bacterial infections may be expected.

Likewise, the widespread use of antibiotics in the community prior to attendance at the hospital (as noted in a quarter of the patients with confirmed BSI) may have led to underrepresentation of more fastidious organisms (e.g. *S. pneumoniae*) and/or the selection of the more resistant organisms, although we did not observe any differences in CSO yield between patients with and without prior antibiotic use. Furthermore, the distinction between community- and hospital-acquired infection was not always crystal clear as patients may not have always mentioned their prior visits to health care centres or private clinics. Therefore our findings may not fully represent the pathogen distribution and resistance patterns in the general population; other methodologies have been suggested for this purpose [14].

In addition, it was not among the present objectives to include media for mycobacterial blood culture nor additional testing for other likely causes of fever in Cambodia e.g. influenza, malaria, leptospirosis, dengue, scrub typhus. In this respect our data complement other research [15] on the causes of fever in Cambodian patients attending first line health services.

Finally, clinical and epidemiological data were not available from all patients and, along the isolation of CSO, we observed a high contamination rate of the blood cultures. As part of a larger capacity-building project, considerable effort was spent to tackle this problem with education and feedback sessions.

In spite of these limitations, we think that our findings are well in line with and are complementing other data from the Southeast Asian region. For instance, blood culture studies from Thailand [16], [17] also described a high prevalence of Gram negative pathogens, with a predominance of non-typhoid *Salmonella* in HIV-positive patients. Besides *B. pseudomallei*, also *Streptococcus suis* [18] and *Salmonella Choleraesuis* [19] appear to be regionally relevant pathogens. In addition, *Acinetobacter* spp. has been described also a common cause of hospital- and community-acquired invasive infections in other Asian settings [20] as well. The relative paucity of *Salmonella* Typhi in our study is probably due to the fact that the hospital has no paediatric services and attracts a somewhat older and comorbid population.

Recent surveillance data from the Asian region [21] show overall high prevalence of ESBL in *Enterobacteriaceae*. The nearly 50% ESBL prevalence among *E. coli* in our study appears well in line with the rates of ESBL–positivity of this pathogen in Thailand (56%) and Vietnam (42.0%). Only in India and China the prevalence of ESBL in *E. coli* was higher (67.1 and 65.4% respectively).

The presence of ESBL in *E. coli* and other *Enterobacteriaceae* in Southeast Asia is a relatively recent phenomenon, its presence and exponentially rising prevalence occurred in the literature around the start of the new millennium [22]. Of note, much lower resistance rates were noted in Laos [23].

As our report provides the first description of ESBL-positive pathogens from systematically collected community-acquired bloodstream infections, it complements the observations of Ruppé and coworkers [24] in 2007 describing the presence of ESBL in 37.7% of *E coli* from 93 urinary samples from patients in Phnom Penh, all of the CTX-M- type. In depth molecular studies of the ESBL-types in the *Enterobacteriaceae* from our study are currently on-going.

So far we did not yet observe carbapenem resistance in *Enterobacteriaceae* in Cambodia, but this may only be a matter of time given the recently found presence of NDM-1 positive *Enterobacteriaceae* in seepage water in Hanoi, Vietnam [25] and in clinical samples in Thailand [26]. In addition, given the high ESBL-rates, carbapenem antibiotics have been recently introduced in the country. Finally, we found carbapenem resistance in nearly 5% of *Acinetobacter* sp., which may provide an additional reservoir of resistance genes.

Our data highlighted also *S. aureus* as an important cause of BSI in Cambodia. This confirms findings from other Southeast Asian countries e.g. Laos and Thailand [27]; the Thai authors observing comparable MRSA rates. Our data on *S. aureus* BSI complement the recent descriptions of MRSA as a cause of community-acquired skin and soft tissue infections in children in Siem Reap (North West Cambodia) [28].

What may be the main drivers of these high resistance rates in Cambodia, a country that has just recovered from many years of civil war and international isolation? However speculative, we assume that the unregulated use of antibiotics in men and livestock plays a pivotal role. While solid usage data for antibiotics in Cambodia are absent so far, there is a large body of published information on poor quality anti-parasitic drugs (in particular antimalarials) in Cambodia and in the Southeast Asian region [29]. Further, a pilot survey in three Cambodian health centres [30] revealed a 66–100% antibiotic prescription rate per consultation, with antibiotic prescriptions being appropriate in as low as 3–45% of cases. Recent agricultural reports from neighbouring Vietnam [31] and Thailand showed long lists of antibiotics used for the prevention and treatment of infections in livestock, including fluoroquinolones, beta-lactams, cephalosporins, macrolides and even actual third line antibiotics such as polymyxins. A recent Cambodian study on 152 chicken carcasses collected at 10 markets across the country revealed high contamination rates with enteric pathogens, many of which with complex resistance patterns [32]. Taken together, these facts suggest intense antibiotic use in humans and animals in the entire region which warrants urgent surveillance and regulation.

Further transmission of selected resistant organisms may then occur at crowded homes with insufficient sanitation or potable water supply, or in health care settings where basic infrastructure and infection control measures are often lacking or not seen as a priority [33]. Nosocomial infection in low-resource settings is probably frequent but underreported [34].

As a response, Cambodia’s Health authorities issued a first National Medicines Policy in Cambodia in 1995; a more extensive Pharmaceutical Sector Strategic Plan was introduced in 2006 with the aim of promoting Good Pharmacy Practice while focusing on access, quality and rational use of drugs [35]. In addition, a comprehensive plan for the improvement of infection control at the health care facilities was issued in 2010 [36].

If confirmed in other settings in the country, the observed high resistance rates seriously jeopardize the treatment options for community-associated BSI in Cambodia. Based on these microbiological data, over one third of individuals with BSI in SHCH would require treatment with broad spectrum antibiotics which are not commonly available in public hospitals in Cambodia, nor are they included in the actual national Essential Drugs’ List (with the exception of low-dose ceftazidime) [37]. In SHCH broad spectrum antibiotics such as ceftazidime, meropenem and vancomycin were obtained via a drug donation program, integrated in the local standard treatment guidelines and their use is monitored. This is however not yet the case for most other health care settings in the country. Given the increased morbidity and mortality associated with the use of ineffective empirical antibiotics for invasive infections with resistant bacteria [2], as also suggested by the elevated and early mortality in our study population, there is an urgent need for sustainable and affordable access to third line antibiotics for the treatment of ESBL-positive *E. coli* and other *Enterobacteriaceae*, melioidosis and invasive MRSA infections in the entire country. Along such an updated essential drugs list, standard treatment guidelines and the medical curriculum require thorough and urgent revision. This is however a lengthy process and holds important financial and organisational implications. In addition, locally adapted antibiotic stewardship and infection control programs should be introduced at the different levels of patient care, while nationwide surveillance of bacterial resistance and antibiotic use is essential for planning and monitoring of these interventions. Ideally, this resistance problem should be addressed and surveyed at a regional level. Finally, the introduction of targeted vaccination e.g. for typhoid fever, *S. pneumoniae* and Haemophilus influenzae type B (HiB) may be a complementary intervention which has proven successful in reducing the number of infections in other low-resource settings [38].

Confirmation of our findings from blood cultures studies in rural hospitals and other patient groups in Cambodia (e.g. paediatric and adolescent patients) is certainly needed as well as community-based surveillance of resistance in healthy carriers. An expansion of solid microbiological capacity in Cambodia is urgently needed to ensure these clinical, research and surveillance activities.

Further research would also include quantitative and qualitative research on the use of antibiotics in the community, health care settings and agriculture and further identification of those most at risk of life-threatening resistant bacterial infections, e.g. the elderly, patients with diabetes or HIV as suggested by our exploratory findings.

### Conclusion

Adult patients with community-acquired BSI in Cambodia are very likely to be infected with highly resistant Gram negative pathogens. These findings warrant intensified microbiological surveillance and should be a an urgent call for concerted nation-wide action to contain bacterial resistance in Cambodia.

## Supporting Information

Document S1 **Ethical approval of ITM’s Institutional Review Board.**
(PDF)

Document S2 **Ethical approval of University of Antwerp.**
(PDF)

Document S3 **Ethical approval of Cambodia National Ethical Committee.**
(PDF)

Document S4 **Vlieghe E et al. Melioidosis, Pnom Penh, Cambodia. Emerg Infect Dis Vol. 17, No. 7, July 2011.**
(PDF)

Document S5 **Vlieghe E et al. Azithromycin and Ciprofloxacin Resistance in Salmonella.** Bloodstream Infections in Cambodian Adults. PloS NTD, 6 (12), e1933.
(PDF)
