# Supplementary material for: Bloodstream Infection among Adults in Phnom Penh, Cambodia: Key Pathogens and Resistance Patterns
Source: PLoS One. 2013 Mar 29;8(3):e59775. doi: 10.1371/journal.pone.0059775 (PMC3612098; doi:10.1371/journal.pone.0059775)
Supplement: Document S1 — Ethical approval of ITM’s Institutional Review Board. (PDF) [file pone.0059775.s001.pdf]

Instituut voor Tropische Geneeskunde
Institut de Médecine Tropicale
Institute of Tropical Medicine
Instituto de Medicina Tropical
Stichting van Openbaar Nut | 0410.057.701

Dr. J. Jacobs
Departement Klinische Wetenschappen

IRB/AB/ec/56

Antwerpen, 7 mei 2008

Geachte collega

Betreft: **Surveillance of antimicrobial resistance among consecutive blood culture isolates in tropical settings**

Ons refnr.: **08 17 2 613**

Na bespreking tijdens de vergadering van de IRB van heden deel ik u mee dat deze studie werd goedgekeurd.

Uw studie zal aan het EC van het UZA bezorgd worden zodat het dossier besproken kan worden op een van hun eerstvolgende vergaderingen.

Met vriendelijke groeten

Prof. A. Buvé
Voorzitster IRB a.i.

Nationalestraat 155, B-2000 Antwerpen - België
Tel: +32 (0)3 247 66 66
Fax: +32 (0)3 216 14 31
Website: http://www.itg.be
E-mail: info@itg.be

**IRB number :**	**08 17 2 613**
**Title project/study:**	**Surveillance of antimicrobial resistance among consecutive blood culture isolates in tropical settings**
**Investigator:**	**Dr. J. Jacobs**<br>**Departement Klinische Wetenschappen**
	**Evaluation/comments on the proposed project/study**
Background and study objectives (rationale and relevance):	OK
Approach for the recruitment of the study subjects:	OK
Study costs:	OK
Study procedures:	OK
Risk evaluation:	OK
Benefit evaluation:	OK
Confidentiality and privacy:	OK
Informed consent:<br>- language<br>- description of the content of the study<br>- foreseeable risks & benefits<br>- confidentiality<br>- privacy<br>- medical treatment<br>- contact information<br>- voluntary participation & refusal<br>- legal representative	OK
Final remarks:	OK
**Conclusion**	**Positive. This study will be submitted to the EC of UZA.**
