# Supplementary material for: Bloodstream Infection among Adults in Phnom Penh, Cambodia: Key Pathogens and Resistance Patterns
Source: PLoS One. 2013 Mar 29;8(3):e59775. doi: 10.1371/journal.pone.0059775 (PMC3612098; doi:10.1371/journal.pone.0059775)
Supplement: Document S2 — Ethical approval of University of Antwerp. (PDF) [file pone.0059775.s002.pdf]

UNIVERSITAIR ZIEKENHUIS ANTWERPEN

kennis / ervaring / zorg

Wilrijkstraat 10
2650 Edegem
Tel. (algemeen): 03 821 30 00
Fax (algemeen): 03 829 05 20
www.uza.be

COMITE VOOR MEDISCHE ETHIEK

VOORZITTER
Prof. dr. Patrick Cras

SECRETARIAAT
tel: 03 821 35 44
fax: 03 821 42 54

Dr. J. JACOBS
Instituut voor Tropische Geneeskunde
Klinische Wetenschappen
2000 Antwerpen

Surveillance of antimicrobial resistance among consecutive blood culture isolates in tropical settings.

datum	ons kenmerk	contactpersoon
9/06/2008	8/20/96	Annelies Van Looy / Kim Vernimmen<br>ethisch.comite@uza.be

DEFINITIEF GUNSTIG ADVIES

Geachte Collega,

Het Ethisch Comité van het Universitair Ziekenhuis Antwerpen bevestigt dat bovenvermelde studie voldoet aan de criteria gesteld in de wet van 7 mei 2004 en geeft een gunstig advies dd. 9/06/2008 .

De volgende bijlagen werden volgens de GCP-ICH richtlijnen door het Ethisch Comité goedgekeurd:

- Begeleidend schrijven versie 20/04/2008
- Protocol Version 080420 AMR ITM JJ versie 20/04/2008

Tenslotte verzoeken wij u ons mee te delen indien een studie niet wordt aangevat, of wanneer ze wordt afgesloten of vroegtijdig onderbroken.

Met vriendelijke groeten,

Prof. dr. G. IEVEN
Ondervoorzitter Ethisch Comité UZ Antwerpen

Cc: FAGG - FAGG - Research & Development Department, Victor Hortaplein 40, bus 40 - 1060 Brussel

Ethisch Comité
Universitair Ziekenhuis Antwerpen
Wilrijkstraat 10
2650 EDEGEM

Tel.: 03.821.35.44
Fax: 03.821.42.54
e-mail : ethisch.comite@uza.be

Samenstelling Ethisch Comité sinds 18 februari 2008

Voorzitter
Prof. dr. P. Cras

Leden aanwezig op vergadering van 9/06/2008

Leden	Functie	Geslacht	Aanwezig
BOSMANS Jean-Louis	Nefroloog	M	☑
CLAEYS Marc	Cardioloog	M	☑
CRAS Patrick	Voorzitter/Neuroloog	M	☐
GERMONPRE Paul	Pneumoloog	M	☑
HERMAN Arnold	Farmacoloog	M	☐
IEVEN Greet	Ondervoorzitter/Klinisch Bioloog	V	☑
JACOBS Werner	Anatomopatholoog	M	☐
MICHIELS Barbara (*)	Huisarts	V	☑
SMETS Erica	Oftalmoloog	V	☑
TEN BROECKE Pieter	Anesthesist	M	☑
VAN BEEUMEN Gerda	Transplant-Coördinator	V	☑
VAN BORTEL Paulus (*)	Filosoof	M	☐
VAN GENECHTEN Nancy	Verpleegkundige	V	☐
VAN REEMPTS Patrick	Neonatoloog	M	☐
VANSWEEVELT Thierry	Jurist	M	☐

(*) niet verbonden aan het Universitair ZiekenhuisAntwerpen.

The Ethics Committee states that no individual member of the Ethics Committee who may have an affiliation with the study or sponsor, has voted in the deliberations for this trial.

The Ethics Committee states that it is organised and operates according to the ICH/GCP guidelines, the applicable laws and regulations, and their own written operating procedures.
