# Supplementary material for: Bloodstream Infection among Adults in Phnom Penh, Cambodia: Key Pathogens and Resistance Patterns
Source: PLoS One. 2013 Mar 29;8(3):e59775. doi: 10.1371/journal.pone.0059775 (PMC3612098; doi:10.1371/journal.pone.0059775)

Melioidosis, Phnom Penh, Cambodia

Erika Vlieghe, Lim Kruy, Birgit De Smet, Chun Kham, Chhun Heng Veng, Thong Phe, Olivier Koole, Sopheak Thai, Lut Lynen, and Jan Jacobs

We describe 58 adult patients with melioidosis in Cambodia (2007–2010). Diabetes was the main risk factor (57%); 67% of infections occurred during the rainy season. Bloodstream infection was present in 67% of patients, which represents 12% of all bloodstream infections. The case-fatality rate was 52% and associated with inappropriate empiric treatment.

Melioidosis, an infectious disease caused by *Burkholderia pseudomallei*, is endemic to Southeast Asia and tropical Australia (*1*,*2*). *B. pseudomallei* is a gram-negative bacterium that causes lung or soft tissue infections with or without bloodstream infection (BSI) (*3*); the case-fatality rate can exceed 80%. Treatment includes third-generation cephalosporins or carbapenems, followed by maintenance courses of sulfamethoxazole/trimethoprim (SMX/TMP) with or without doxycycline.

In Cambodia, few microbiologically confirmed cases have been described (*4*–*7*). We describe 58 adult patients in whom melioidosis was diagnosed during July 1, 2007–January 31, 2010, at Sihanouk Hospital Centre of Hope, Phnom Penh, Cambodia.

The Study

Melioidosis was defined as growth of *B. pseudomallei* from any clinical specimen (blood, pus, or urine). Nonfermentative gram-negative rods suspected for *B. pseudomallei* (wrinkled colonies, oxidase positive, polymyxin and gentamicin resistant, amoxicillin/clavulanic acid susceptible [*8*]) were identified by the API 20NE system (bioMérieux, Marcy L'Etoile, France). MICs were determined with Etest (Biodisk, Solna, Sweden). Interpretive criteria were those defined for *B. pseudomallei* by the Clinical and Laboratory Standards Institute (*9*).

Recurrences were defined as the culture-confirmed reappearance of symptoms after initial response to therapy (*10*). Treatment was considered appropriate if it contained ceftazidime, a carbapenem, or amoxicillin/clavulanic acid with or without SMX/TMP.

Risk factors were assessed by univariate analysis. Ethical approval was granted by the University Hospital Antwerp and the National Ethical Committee in Phnom Penh.

Seventy-one isolates of *B. pseudomallei* were recovered from 58 patients (mean age 49 years, range 18–73 years); 34 (59%) were men. Seasonal patterns of infection are shown in Figure 1 and geographic distribution of patients' homes (56) in Figure 2. Melioidosis was diagnosed in 39 (67%) patients during the rainy season. In 39 patients, *B. pseudomallei* was recovered from blood samples, which represented 12.0% of the 328 clinically significant organisms from BSIs and 1.0% of the 3,976 systemic inflammatory response syndrome episodes during the study. In 2 patients, melioidosis was retrospectively considered a recurrence 137 and 231 days postinfection.

Fifty-four (52 initial and 2 successive) isolates were used for resistance testing (Table 1). No resistance was noted for ceftazidime, meropenem, amoxicillin/clavulanic acid or doxycycline, but 12 (22.2%) isolates had MICs equal to the susceptibility breakpoint for chloramphenicol.

Risk factor information available for 51 patients included diabetes mellitus (34 [59%] patients); alcoholism (7 [12%]); and corticosteroid use (3 [5%]). Most (39) patients had BSI with or without pneumonia. Median delay to growth of blood cultures was 4 days (range 2–8). During the study, *B. pseudomallei* was increasingly recovered from nonblood specimens, in line with growing laboratory expertise. Involvement of the lungs was noted in 28 (48%) patients. Other sites included skin and soft tissue (17 patients), bone and joints (8), urogenital tract (4), spleen (8), liver (5), and psoas muscle and thyroid gland (1 each). Infection was often multifocal. Seventeen (29%) patients had shock or multiorgan failure. The median delay from symptom onset to seeking treatment was 28 days (range 1–730 days).

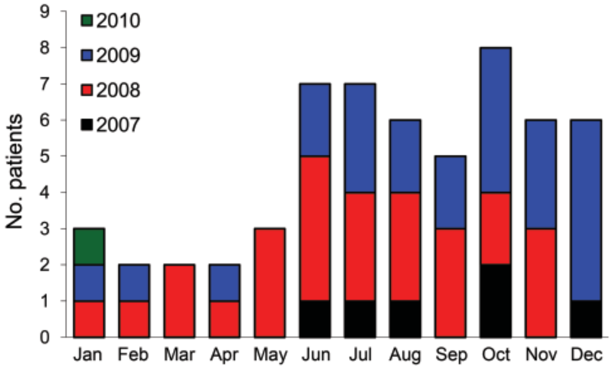

Figure 1. Number of patients in whom melioidosis was diagnosed, by season, Phnom Penh, Cambodia, July 1, 2007–January 31, 2010.

Author affiliations: Institute of Tropical Medicine, Antwerp, Belgium (E. Vlieghe, B. DeSmet, O. Koole, L. Lynen, J. Jacobs); and Sihanouk Hospital Centre of Hope Phnom Penh, Cambodia (L. Kruy, C. Kham, C.H. Veng, T. Phe, S. Thai)

DOI: 10.3201/eid1707.101069

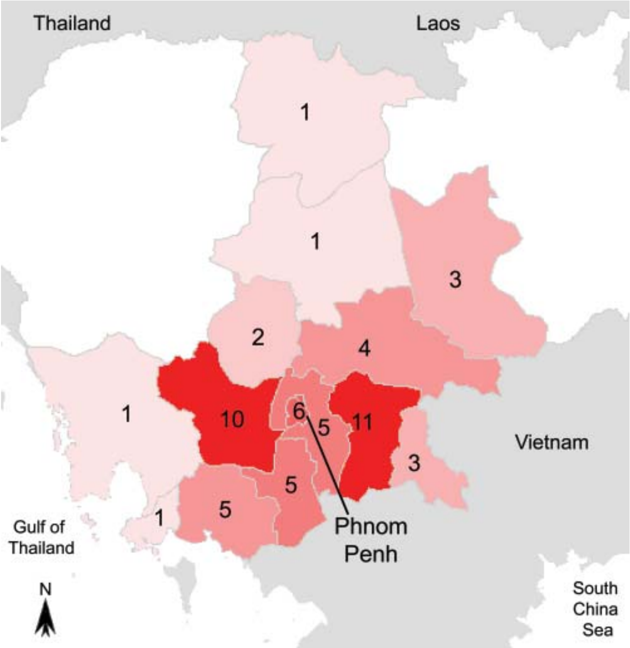

Figure 2. Map of Cambodia with geographic origin of the 58 patients with melioidosis diagnosed during July 1, 2007–January 31, 2010.

Thirty (52%) patients died; no outcome data were available for 3 patients. Death occurred early; 19 (63%) nonsurvivors died within 1 week after admission. In univariate analysis, risk factors for death were signs of shock, multiorgan failure, or BSI and not receiving appropriate empiric therapy (Table 2). Among the 25 survivors, 22 (88%) recovered without recurrence; the other 3 were lost to follow-up during maintenance treatment. The mean duration of follow-up was 12.8 months (range 3.5–28 months).

Treatment data were available for 53 patients; 18 (34%) received inappropriate empiric therapy; all died early. Thirty-five patients received appropriate treatment; 23 patients were given ceftazidime (2 g 3×/d for ≥14 days) with or without SMX/TMP (30 mg/kg 2×/d), 6 received amoxicillin–clavulanic acid (875–1,000 mg 3×/d) with or without SMX/TMP, and another 6 received SMX/TMP with doxycycline (200 mg 1×/d). Twenty-three patients continued maintenance therapy, primarily SMX/TMP with or without doxycycline (22 patients). Total treatment duration ranged from 3 to 6 months.

Conclusions

Our findings of melioidosis in 58 adults complement the recently published data on melioidosis in children in Cambodia (*7*). A limitation of our study is its retrospective nature; a small number of patients precluded detailed study of risk factors and calculation of population-based incidence data. In addition, we have not yet studied the isolates to the genetic level. However, presently used phenotypic characteristics have been validated against molecular reference standards as accurate tools for *B. pseudomallei* identification (*8*).

Risk factors for patients and epidemiologic profiles were similar to those observed in northeastern Thailand (*1*,*11*). Most cases occurred during or shortly after the rainy season (May–November); diabetes mellitus was the most relevant risk factor, which is consistent with findings from other regions where melioidosis is endemic (*1*,*11*,*12*). Diabetes is quickly emerging in Cambodia and remains a difficult-to-treat chronic disease in poor rural settings (*13*).

In this study, nearly two thirds of patients had BSI and half had pneumonia. These data are consistent with studies from Thailand and Australia, where BSI and pneumonia accounted for 46%–60% and 50%–60% of manifestations, respectively (*11*,*12*). Soft tissue and deep organ abscesses were also frequent. The finding of a spleen abscess in a melioidosis-endemic area should trigger suspicion of melioidosis.

In our study, distinguishing primary infection or reinfection from relapse was not possible, but the seasonal link suggests recent infections. The 2 recurrences in our study were probably relapses caused by insufficient

Table 1. MICs for 54 *Burkholderia pseudomallei* isolates, Phnom Penh, Cambodia, July 1, 2007–January 31, 2010*

	MIC, µg/mL															Breakpoints, µg/mL	
Antimicrobial drug	0.38	0.5	0.75	1	1.5	2	3	4	6	8				MIC_{50}	MIC_{90}	S	R
Meropenem	3	29	16	1	3	2	–	–	–	–				0.5	1.5	≤4	≥16
Doxycycline†	–	13	19	18	3	1	–	–	–	–				0.75	1	≤4	≥16
Ceftazidime†	–	2	0	18	25	7	2	–	–	–				1.5	2	≤8	≥32
Amoxicillin/ clavulanic acid	–	1	0	13	30	7	2	1	–	–				1.5	2	≤8	≥32
Chloramphenicol	–	–	–	–	1	1	0	17	16	12				6	8	≤8	≥32
	0.032	0.038	0.047	0.064	0.094	0.125	0.19	0.25	0.38	0.75	1	1.5	3				
Sulfamethoxazole/ trimethoprim	3	1	7	12	5	9	4	7	1	1	3	1	0	0.125	0.75	≤2	≥4

*MICs determined by Etest (AB Biodisk, Solna, Sweden). MIC_{50}, MIC for 50% of isolates; MIC_{90}, MIC for 90% of isolates; S, susceptible; R, resistant; –, no isolates with this MIC.
†53 isolates included.

Table 2. Predictors of death for 55 patients with melioidosis, Phnom Penh, Cambodia, July 1, 2007–January 31, 2010*

Risk factor	Presence of risk factor	No. patients	No. patients who died	Relative risk (95% CI)	p value
Age >55 y	Y	24	14	1.13 (0.70–1.83)	0.786
	N	31	16		
Male sex	Y	31	18	1.16 (0.70–1.91)	0.595
	N	24	12		
Rainy season	Y	36	23	1.73 (0.92–3.28)	0.087
	N	19	7		
Diabetes	Y	32	14	0.70 (0.41–1.21)	0.359
	N	16	10		
Alcoholism	Y	7	6	0.97 (1.19–3.22)	0.092
	N	32	14		
Clinical sign					
Duration of symptoms <2 mo	Y	12	3	2.26 (0.80–6.42)	0.152
	N	23	13		
Bloodstream infection	Y	37	28	6.81 (1.82–25.50)	**<0.001**
	N	18	2		
Pneumonia	Y	28	18	1.52 (0.90–2.57)	0.172
	N	26	11		
Deep abscesses	Y	15	6	0.80 (0.38–1.67)	0.742
	N	24	12		
Bone/joint infection	Y	8	4	1.04 (0.47–2.28)	1.000
	N	29	14		
Urogenital infection	Y	5	1	0.38 (0.64–2.25)	0.345
	N	38	20		
Skin and soft tissue infection	Y	19	6	0.48 (0.24–0.97)	**0.023**
	N	35	23		
Shock or multiorgan failure	Y	17	13	4.59 (1.60–13.32)	**<0.001**
	N	18	3		
Therapy					
Inappropriate empiric therapy	Y	18	18	3.50 (2.07–5.90)	**<0.001**
	N	35	10		

*Not all information about outcome predictors was available from all patients. Fisher exact test was used for categorical variables and Student *t* test for continuous variables. CI, confidence interval. Statistically significant associations (p<0.05) are shown in **boldface**.

treatment of the first (unrecognized) episode, as has been described in patients in Thailand and Australia (*10*). The possibility of relapse emphasizes the need for intense follow-up during and after the treatment course.

We noted a high case-fatality rate, especially among patients with BSI or pneumonia, who were in shock or had multiorgan failure, or who were receiving inappropriate empirical therapy. Potential interventions to decrease risk factors for death caused by melioidosis include improved sepsis care and ensured availability of effective drugs such as ceftazidime, carbapenems, and amoxicillin/clavulanic acid. Although we did not demonstrate resistance to any of these antimicrobial drugs, resistance can occur during therapy; follow-up blood cultures during treatment is essential (*14*).

During the 19-month study, we observed a learning curve on melioidosis at several levels in the hospital. Even though melioidosis is well known in the Southeast Asian region (*2*), it was unfamiliar to most clinicians and laboratory staff at the start of the study. Our findings may also have an effect at the national level, especially regarding early diagnosis and treatment. Awareness must be raised among health care workers and high-risk patient groups (e.g., diabetes patients). Development of quality-assured and affordable microbiological capacity throughout the country is also crucial in the broader picture of surveillance and containment of antimicrobial drug resistance. Careful adaption of local treatment guidelines is essential and has been successful in other settings, e.g., Northern Territory, Australia (*15*). Because melioidosis appears endemic to Cambodia, the public health impact of this disease warrants further research and action.

The Bloodstream Infection Surveillance Project was supported by Project 2.08 of the third framework agreement between the Belgian Directorate General of Development Cooperation and the Institute of Tropical Medicine, Antwerp, Belgium.

Dr Vlieghe is an infectious disease physician at the Department of Clinical Sciences, Institute of Tropical Medicine (Antwerp, Belgium) and the Department of Tropical Medicine,

University Hospital, Antwerp. Her research interests include bacterial infections and antimicrobial drug resistance in tropical low-resource settings.

References

1. Chaowagul W, White NJ, Dance DA, Wattanagoon Y, Naigowit P, Davis TM, et al. Melioidosis: a major cause of community-acquired septicemia in northeastern Thailand. J Infect Dis. 1989;159:890–9. doi:10.1093/infdis/159.5.890
2. Currie BJ, Dance DA, Cheng AC. The global distribution of *Burkholderia pseudomallei* and melioidosis: an update. Trans R Soc Trop Med Hyg. 2008;102(Suppl 1):S1–4. doi:10.1016/S0035-9203(08)70002-6
3. White NJ. Melioidosis. Lancet. 2003;361:1715–22. doi:10.1016/S0140-6736(03)13374-0
4. Overtoom R, Khieu V, Hem S, Cavailler P, Te V, Chan S, et al. A first report of pulmonary melioidosis in Cambodia. Trans R Soc Trop Med Hyg. 2008;102(Suppl 1):S21–5. doi:10.1016/S0035-9203(08)70007-5
5. Scully RE, Mark EJ, McNeely WF, McNeely BU. Case records of the Massachusetts General Hospital. Weekly clinicopathological exercises. Case 40-1992. A 43-year-old Cambodian man with several years of recurrent bouts of fever and abdominal pain. N Engl J Med. 1992;327:1081–7. doi:10.1056/NEJM199210083271508
6. Chan CK, Hyland RH, Leers WD, Hutcheon MA, Chang D. Pleuropulmonary melioidosis in a Cambodian refugee. Can Med Assoc J. 1984;131:1365–7.
7. Pagnarith Y, Kumar V, Thaipadungpanit J, Wuthiekanun V, Amornchai P, Sin L, et al. Emergence of pediatric melioidosis in Siem Reap, Cambodia. Am J Trop Med Hyg. 2010;82:1106–12. doi:10.4269/ajtmh.2010.10-0030
8. Hodgson K, Engler C, Govan B, Ketheesan N, Norton R. Comparison of routine bench and molecular diagnostic methods in identification of *Burkholderia pseudomallei*. J Clin Microbiol. 2009;47:1578–80. doi:10.1128/JCM.02507-08
9. Clinical and Laboratory Standards Institute. Performance standards for antimicrobial susceptibility testing—twentieth informational supplement, approved standard M100-S20. Wayne (PA): The Institute; 2010.
10. Limmathurotsakul D, Chaowagul W, Chierakul W, Stepniewska K, Maharjan B, Wuthiekanun V, et al. Risk factors for recurrent melioidosis in northeast Thailand. Clin Infect Dis. 2006;43:979–86. doi:10.1086/507632
11. Suputtamongkol Y, Hall AJ, Dance DA, Chaowagul W, Rajchanuvong A, Smith MD, et al. The epidemiology of melioidosis in Ubon Ratchatani, northeast Thailand. Int J Epidemiol. 1994;23:1082–90. doi:10.1093/ije/23.5.1082
12. Currie BJ, Fisher DA, Howard DM, Burrow JN, Lo D, Selva-Nayagam S, et al. Endemic melioidosis in tropical northern Australia: a 10-year prospective study and review of the literature. Clin Infect Dis. 2000;31:981–6. doi:10.1086/318116
13. King H, Keuky L, Seng S, Khun T, Roglic G, Pinget M. Diabetes and associated disorders in Cambodia: two epidemiological surveys. Lancet. 2005;366:1633–9. doi:10.1016/S0140-6736(05)67662-3
14. Dance DA, Wuthiekanun V, Chaowagul W, White NJ. Interactions in vitro between agents used to treat melioidosis. J Antimicrob Chemother. 1989;24:311–6. doi:10.1093/jac/24.3.311
15. Elliott JH, Anstey NM, Jacups SP, Fisher DA, Currie BJ. Community-acquired pneumonia in northern Australia: low mortality in a tropical region using locally-developed treatment guidelines. Int J Infect Dis. 2005;9:15–20. doi:10.1016/j.ijid.2004.09.008

Address for correspondence: Erika Vlieghe, Department of Clinical Sciences, Institute of Tropical Medicine, Nationalestraat 155, 2000 Antwerp, Belgium; email: evlieghe@itg.be

The opinions expressed by authors contributing to this journal do not necessarily reflect the opinions of the Centers for Disease Control and Prevention or the institutions with which the authors are affiliated.

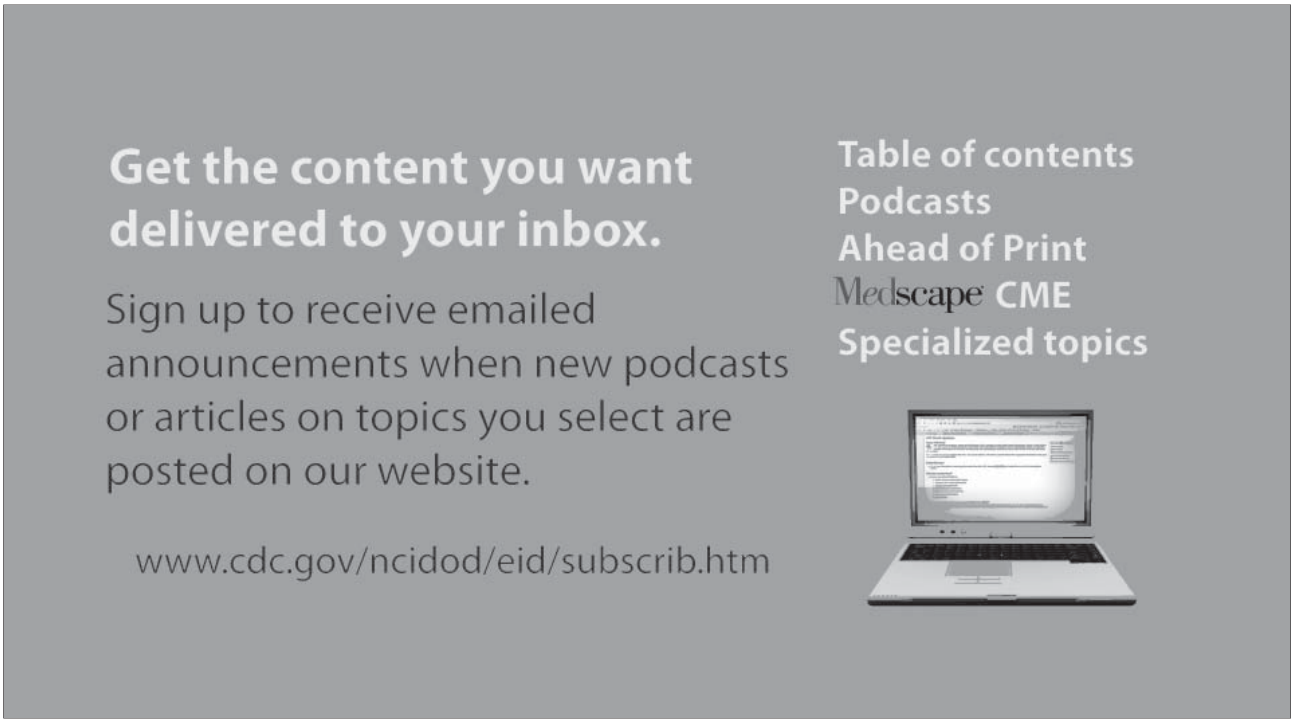

Supplement: Document S4 — Vlieghe E et al. Melioidosis, Pnom Penh, Cambodia. Emerg Infect Dis Vol. 17, No. 7, July 2011. (PDF) [file pone.0059775.s004.pdf]
