# Supplementary material for: Bloodstream Infection among Adults in Phnom Penh, Cambodia: Key Pathogens and Resistance Patterns
Source: PLoS One. 2013 Mar 29;8(3):e59775. doi: 10.1371/journal.pone.0059775 (PMC3612098; doi:10.1371/journal.pone.0059775)
Supplement: Document S3 — Ethical approval of Cambodia National Ethical Committee. (PDF) [file pone.0059775.s003.pdf]

ក្រសួងសុខាភិបាល
MINISTRY OF HEALTH
គណៈកម្មាធិការជាតិក្រមសីលធម៌
សំរាប់ការស្រាវជ្រាវសុខភាពដែលទាក់ទងនឹងមនុស្ស
National Ethics Committee for Health Research

ព្រះរាជាណាចក្រកម្ពុជា
KINGDOM OF CAMBODIA
ជាតិ សាសនា ព្រះមហាក្សត្រ
NATION RELIGION KING

លេខ....021 NECHR

រាជធានីភ្នំពេញ, ថ្ងៃទី 20 ខែ 02 ឆ្នាំ2012

Dr. Phe Thong

Project: Request for continuation and amendment of the protocol entitled "Surveillance of antimicrobial resistance among consecutive culture isolates in tropical settings. Version N° 2.0, dated 20th January, 2012"

Reference: 17th February, 2012 NECHR meeting minute

Dear Dr. Phe Thong,

I am pleased to inform you that your request for continuation and amendment of your study protocol entitled "Surveillance of antimicrobial resistance among consecutive culture isolates in tropical settings. Version N° 2.0, dated 20th January, 2012" has been approved by National Ethic Committee for Health Research (NECHR) in the meeting on 17th February, 2012. This approval is valid for twelve months after the approval date.

The Principal Investigator of the project shall submit following document to the committee's secretariat at the National Institute of Public Health at #2 Kim Il Sung Blvd, Khan Tuol Kok, Phnom Penh. (Tel: 855-23-880345, Fax: 855-23-881949):

- Annual progress report
- Final scientific report
- Patient/participant feedback (if any)
- Analyzing serious adverse events report (if applicable)

The Principal Investigator should be aware that there might be site monitoring visits at any time from NECHR team during the project implementation and should provide full cooperation to the team.

Regards,

Chairman

Prof. ENG HUOT

លេខ ២ មហាវិថី គីម អ៊ិលស៊ុង . ខ័ណ្ឌទួលគោក . រាជធានីភ្នំពេញ . ទូរស័ព្ទ (៨៥៥-២៣) ៨៨០ ៣៤៥ . ទូរស័ព្ទដៃ (៨៥៥-១២) ២៨០ ៧៩០ . (៨៥៥-១២) ៨៤២ ៤៤២
2 Blvd KIM YL SUNG, Khan Toul Kork, Phnom Penh , Tel : (855-23) 880 345 , Mobile phone : (855-12) 280 790, (855-12) 842 442
